# Morphological and quantitative study of the inferior alveolar nerve canal in hemifacial microsomia

**DOI:** 10.1038/s41598-024-54318-z

**Published:** 2024-02-27

**Authors:** Xin Li, Xiaojun Chen, Ziwei Zhang, Xuetong Wang, Wenqing Han, Byeong Seop Kim, Yingjie Yan, Gang Chai, Yan Zhang

**Affiliations:** Department of Plastic and Reconstructive Surgery, Shanghai Ninth People’s Hospital, Shanghai Jiao Tong University School of Medicine, 639 Zhi Zao Ju Road, Shanghai, 200011 People’s Republic of China

**Keywords:** Oral diseases, Disability, Oral manifestations

## Abstract

This study aimed to probe into the anatomic course of inferior alveolar nerve canal (IANC) in hemifacial microsomia (HFM) on a large scale, morphological observations and further quantitative study were performed. Patients were classified by Pruzansky–Kaban classification. The anatomic course of IANC was analyzed morphologically with three-dimensional (3D) imaging software among 248 patients. Seven distances between fixed landmarks on both sides were measured for 236 patients. The differences between affected and unaffected sides were compared. Significant differences were found in the entrance (*P* < 0.001), route (*P* < 0.001), and exit (*P* < 0.05) of IANC in type IIb and III HFM. The higher the degree of mandibular deformity was, the higher the incidence of IANC variation was (*P* < 0.05). The distances in the horizontal aspect of IANC including from mandibular foramen to mental foramen (*P* < 0.05) and from mental foramen to gonion (*P* < 0.05) were significantly shorter on the affected side. Abnormalities of the anatomical course of IANC exist in patients with Pruzansky–Kaban type IIb and type III HFM. The reduction of IANC on the affected side in the horizontal distance is more obvious. Three-dimensional imaging assessment is recommended before surgery.

## Introduction

Hemifacial microsomia (HFM) is a congenital cranio-maxillofacial deformities characterized mainly by unilateral mandible anomalies, often accompanied by other ipsilateral deformities, such as microtia, orbital deformities, and soft tissue morbidity, et al., while 5 percent to 30 percent patients have bilateral deformities^1^. Pruzansky–Kaban classification is one of the most extensively used classifications of HFM^2,3^, which provides a basis for stratifying and determining the severity of HFM. It is well known that distraction osteogenesis plays a crucial role in treating HFM^4,5^, as well as ramus and condylar process reconstruction^6^. However, the injury of the inferior alveolar nerve (IAN) sometimes occurs during the operations such as cutting the middle of the ramus, sagittal splitting of the mandible, and titanium plate fixation^7–9^.

The position of the inferior alveolar nerve canal (IANC) has been analyzed by using mandibles of the cadaver, panoramic tomography, cone beam CT, spiral CT scan, and 3D reconstruction^10–12^. Knowledge of the anatomical course of the IANC and its relationship with the fixed landmarks provides important information for safe procedures during operations.

Several researchers have studied the variabilities of the anatomical course of the IANC morphologically or quantitatively^13–17^, ﻿but they have failed to reach a consensus. This study aimed to probe into the anatomical course of the IANC in HFM on a large scale, so as to verify the impairment status of the IANC with HFM.

## Results

### Participants

The information of the patients in the morphological observation was as follows: 143 males and 105 females; age range 6 weeks–26 years; 126 left-sided and 122 right-sided; 20 Pruzansky–Kaban type I, 103 type IIa, 78 type IIb, and 47 type III, while the grouping information of patients in the quantitative measurements was listed in (Table 1).

**Table 1:** The grouping information of patients in the quantitative study.

Classification group	Age (year)/number of the patients					Total
	< 1	1–3	4–6	7–12	> 12	
Low-grade	27	27	26	30	9	119
High-grade	28	35	29	20	5	117
Total	55	62	55	50	14	236

### Morphological observation of the IANC

All the entrances, routes, and exits of IANC in type I and IIa were normal. In regard to type IIb, the numbers of the abnormal entrances, routes, and exits were 3, 11, and 1, respectively. As for type III, the numbers were 13, 17, and 3, respectively. Table 2 listed the detailed results.

**Table 2:** Morphological variabilities of the entrance, route, and exit of the IANC in four types.

Type	Entrance			Route				Exit			
	Nor.^a^	Top^b^	Buccal	Nor.^a^	BMC^c^	Buccal	Anter.^d^	Nor.^a^	Anter.^d^	Absent	Lingual
I	20	0	0	20	0	0	0	20	0	0	0
IIa	103	0	0	103	0	0	0	103	0	0	0
IIb	75	2	1	67	1	3	7	77	0	0	1
III	34	7	6	30	3	10	4	44	1	1	1
Percentage^e^(IIb)	3.85%			14.10%				1.28%			
Percentage(III)	27.66%			36.17%				6.38%			
*P* value^f^	< 0.001			< 0.001				0.035			

^a^Normal position, ^b^On the top of the ramus, ^c^Bifid mandibular canal, ^d^Anterior aspect of the ramus;

^e^Percentage of the abnormal positions;

^f^The *P* values are from two-sided Fisher exact test between type IIb and type III.

With the two-sided Fisher exact test, there were significant differences in the entrances (*P* < 0.001), routes (*P* < 0.001), and exits (*P* < 0.05) of the IANC between patients with type IIa and III HFM. Cochran-Armitage trend test presented that the variation rates of the entrance, route, and exit (Z = 2.6001, P < 0.05) were positively correlated with the severity of the mandibular deformity. That is, those who had severe mandibular deformity could have a higher variation rate of the IANC.

### Quantitative measurements of seven distances

﻿﻿Twelve patients with the absence of mandibular ramus were excluded for the quantitative study. The means of the differences between normal and microsomic sides were calculated (95% confidence interval, in millimeters) using descriptive analysis (Tables 3 and 4). There were no significant differences in the distances Sd, MaF-Go, and MeF-Sym between the unaffected and the microsomic sides in all patients (*P* > 0.05).

**Table 3:** Results of the statistical analysis of the low-grade HFM patients.

Age	﻿Mean differences (mm) (normal side minus affected side) (Mean ± SD)						
	Sd	MaF-Go	MaF-SN	MaF-MeF	MeF-Go	MeF-SN	MeF-Sym
A	0.25 ± 2.40	-0.44 ± 1.57	0.20 ± 1.21	1.96 ± 2.94*	1.85 ± 3.95*	0.03 ± 3.06	0.44 ± 1.71
B	-0.01 ± 1.96	-0.37 ± 2.02	-0.35 ± 2.14	3.28 ± 4.80*	2.54 ± 3.82*	1.14 ± 4.36	0.43 ± 1.67
C	-0.48 ± 3.27	0.21 ± 2.51	0.81 ± 2.55	3.75 ± 5.28*	2.27 ± 4.11*	0.97 ± 6.27	0.11 ± 1.72
D	0.56 ± 3.13	-0.47 ± 2.31	1.77 ± 2.77*	3.66 ± 4.57*	4.99 ± 4.89*	2.26 ± 4.56*	0.62 ± 2.11
E	1.66 ± 5.41	3.50 ± 4.75	3.44 ± 3.59*	3.14 ± 2.75*	3.20 ± 3.27*	6.11 ± 6.86*	0.37 ± 2.65

*Significant difference between normal and microsomic sides from Student *t*-test (*P* < 0.05).

**Table 4:** Results of the statistical analysis of the high-grade HFM patients.

Age	﻿ Mean differences (mm) (normal side minus affected side) (Mean ± SD)						
	Sd	MaF-Go	MaF-SN	MaF-MeF	MeF-Go	MeF-SN	MeF-Sym
A	-0.78 ± 2.36	-0.19 ± 1.33	1.74 ± 2.66*	3.58 ± 3.52*	3.33 ± 3.80*	4.42 ± 3.98*	0.84 ± 2.53
B	-0.51 ± 2.31	-0.07 ± 2.42	1.16 ± 2.89*	6.00 ± 4.39*	5.31 ± 3.85*	4.61 ± 5.50*	0.22 ± 1.39
C	-0.62 ± 3.41	0.82 ± 2.89	2.60 ± 3.72*	5.12 ± 4.56*	4.63 ± 4.78*	4.95 ± 5.30*	0.25 ± 2.97
D	-0.12 ± 3.23	-0.35 ± 2.08	3.20 ± 3.78*	6.96 ± 6.91*	7.12 ± 6.12*	6.40 ± 6.75*	1.20 ± 3.03
E	3.53 ± 6.54	2.27 ± 10.95	10.02 ± 5.74*	4.76 ± 3.61*	13.26 ± 7.28*	11.96 ± 7.83*	1.02 ± 5.59

*Significant difference between normal and microsomic sides from Student *t*-test (*P* < 0.05).

In all low-grade patients, the distances MaF-MeF, and MeF-Go on the microsomic side were significantly shorter than that on the normal side (*P* < 0.05). However, the distances MaF-SN, and MeF-SN were significantly longer on the unaffected side only among the age group D and E (*P* < 0.05).

The distances MaF-SN, MaF-MeF, MeF-Go, and MeF-SN were significantly shorter on the affected side than on the normal side in the high-grade patients of all age groups (*P* < 0.05).

## Discussion

The study's investigation into the anatomic course of the IANC in individuals with HFM sheds light on the intricate relationship between craniofacial abnormalities and neuroanatomy. Several morphological or quantitative studies about the variability of the IANC in HFM had been processed, but rare studies adopted both qualitative and quantitative methods together. We studied the anatomical course of the IANC in HFM morphologically and quantitatively in a larger sample size level by using 3D reconstruction, which helped us to correlate the course of the IANC with the Pruzansky–Kaban classification. Better understanding of the anatomic course of the IANC during the preoperative plan in patients with HFM can help surgeons to carry out successful local anesthetic blocks and surgical procedures.

We analyzed 248 patients for morphological observation, and the findings of the present study were generally consistent with previous studies on variability types^16,17^, no obvious abnormalities of the IANC were observed for patients with HFM. We included patients in both low-grade and high-grade, compared with those studies aimed at low-grade patients^13^, we pay more attention to the patients with higher abnormality rate. We found the frequencies of variability on the entrance, route, and exit in type IIb and type III of our study were not as high as Liu et al.'s study^17^. The discrepancies in sample size and the proportion of the patient types could be the possible reason for this. Besides, several studies used Pruzansky classification only^14,15^, but Kaban modification helps classify patients precisely according to the morphology of the ramus and the condyle, we switched to the Pruzansky–Kaban classification to further find the differences of the course were mainly in type IIb, while there was no difference in IIa, which was significant for clinical decision.

Variations of the mandibular foramen have been reported in hemifacial microsomia, Kan et al^13^. and Das and Suri^18^ have reported mandibular foramina, leading to separate mandibular canal on the medial surface of the mandible, so a bifid or trifid mandibular canal has been revealed. The variation rate of the IANC exit was not as high as that of the entrance and route. While the changes in exit mainly affect the position of the mental nerve^19^. In the surgery involving the mentum of HFM patients, the position of IANC exit should be detected first. Furthermore, HFM patients with type III had a higher frequency of entrance, route, and exit deviation compared with those with type IIb.

Our study first included the quantitative data of HFM from children under twelve months of age and first stratified patients according to age, which could help to reduce age-related errors. Seven distances were measured in our quantitative part, in all patients, there was no significant difference between two sides in the distances MaF-Go, which reflected the relative length of the IAN in the ramus in the vertical aspect. We brought into correspondence with Neiva et al^15^. on the conclusion that no difference in the distance MeF-Sym, thus with high probability, the distance between the intersection point of the midsagittal plane and lower border of the mandible and the mental foramen was longer on the affected side. The visible difference between the normal and microsomic sides in all patients appeared to lie in the part of the horizontal length of the bony canal, which was reflected indirectly by the distances MeF-Go and MaF-MeF, and the finding differed from the previous study^13^, it might be related to the supplement of high-grade patients and the larger sample size. Thus, the length of the IANC on the affected side was shorter, which can be proved by the morphological observations.

It was interesting to note that the differences in distance MaF-SN and MeF-SN showed age-related characteristics in low-grade HFM, the significant differences existed among the patients older than six years of age in the low-grade HFM and all the high-graded patients. We proposed that it might be related to mandibular osteogenesis. The condylar cartilage persisted until 20 years after birth, maintaining the increasing height of the mandible ramus^20^, it happened that age groups D and E included the pubertal stage, which was a period of mandible development, resulting in a difference in patients who were in low -grade group with less severe mandibular deformity. It is also a reminder that more attention could be focused on high-grade patients and low-grade patients over the age of 6 years old during preoperative plan.

The alignment of the IANC on the affected side could not be traced in two patients in this study, which have been excluded. From an embryonic developmental point of view, the anatomical location of the IAN is closely related to mandibular osteogenesis. Before the 6th week of embryonic life, an ossification centre appears lateral to the Meckel cartilage formed by the mandibular protuberance of the first parotid arch, towards the anterior, posterior and lateral sides of the IAN to form the medial and lateral bony plates of the body of the mandible as well as the IANC^16,21^. Therefore, it is possible that during the developmental ossification of the mandible, the bone plates and IANC were not formed on both sides of the IAN, and the IAN may have travelled in the connective or muscular tissue outside of the mandible and was not completely surrounded by the bony structures, and thus the IANC could not be traced on the CT images.

This study has some limitations. The number of inclusive patients was not big enough to ensure a large enough sample size for each type and get each type close in number at the same time. In an ideal situation, there should be more subjects, of a similar age, to reduce the influence of age. What’s more, more series of reference points and distances should be included, such as the relationship between the angle and Sd. However, whether type I and IIa are completely free of IANC variability, and whether there are other undiscovered abnormalities, more center clinical studies are still needed to solve the problems.

In this study, abnormalities of the anatomical course of the IANC exist in patients with Pruzansky–Kaban type IIb and type III HFM, whereas not in type I and IIa. HFM with severe mandibular deformities could have a higher rate of IANC variability. What’s more, the reduction of the IANC on the affected side in the horizontal distance is more obvious than that in the vertical distance. Imaging assessment before surgical intervention could be helpful to avoid IAN damage, especially in patients with type IIb and III.

## Methods

### Subjects

﻿This study was approved by the Institutional Review Board of Shanghai Ninth People’s Hospital. For the morphological study, patients with HFM who were treated at the Department of Plastic and Reconstructive Surgery, Shanghai Ninth People's Hospital from 2014 to 2021 and met the inclusion criteria were enrolled. Inclusion criteria were as follows: diagnosed with unilateral HFM; no previous craniomaxillofacial surgical treatments; and full craniofacial CT scans. Exclusion criteria were as follows: IANC could not be traced on the CT image; combined with other craniofacial deformities/syndromes. Patients from the above group with the presence of ramus were selected for the following quantitative study. They were divided into low-grade (type I, type IIa) and high-grade (type IIb, type III) HFM groups according to the Pruzansky–Kaban classification^22,23^, and meanwhile A (< 1 year), B (1–3 years), C (4–6 years), D (7–12 years), and E (> 12 years) groups according to their ages.

### Image acquisition

All the CT images in DICOM format were imported into Mimics 19.0 software for 3D image reconstruction. ﻿Soft tissues were excluded by using the Thresholding tool (CT value: 226-2976HU), then we obtained the Mask of the skull; the Edit Masks and the Region Growing functions were used to extract the mandible on the coronal, cross-section, and sagittal plane, and we got the complete 3D image of the mandible; then the Nerve tool was used to mark and outline the entrance, route, and exit of bilateral IANC. Finally, the transparency of the 3D image of the mandible was improved to overview the general anatomical course of IANC.

### Data measurement

#### Morphological observation

**Figure 1:**
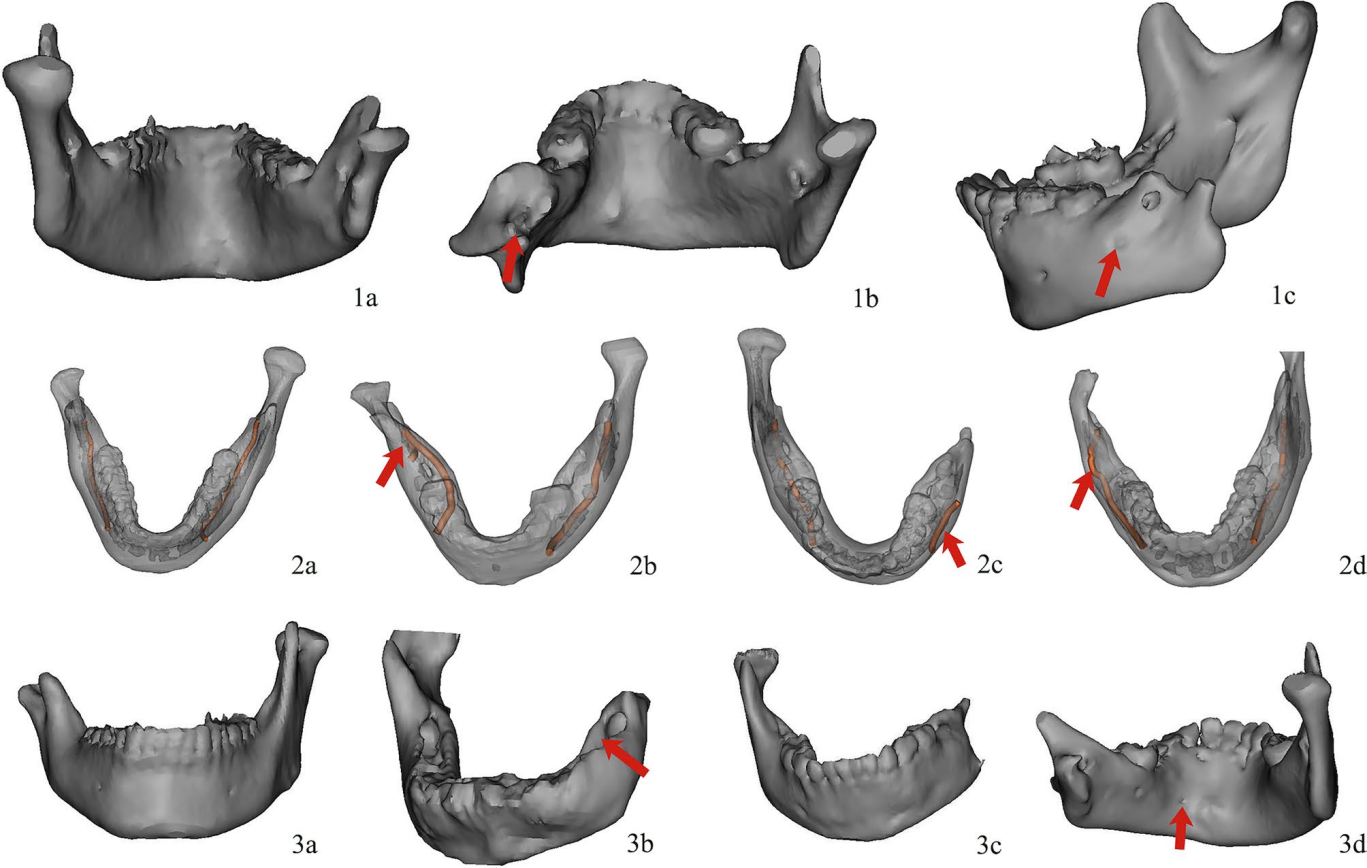
Entrance, route, and exit of the inferior alveolar nerve canal in patients with HFM in 3-dimensional reconstructed models (Red arrows indicate the variabilities on the affected sides) (**a**), Normal entrance. (**b**), Entrance on the top of the ramus. (**c**), Buccal entrance. (a), Normal route. 2b, Bifid mandibular canal. 2c, Buccal side. 2d, Anterior aspect of the ramus. 3a, Normal exit. 3b, Anterior aspect of the ramus. 3c, Absent. 3d, Lingual side.

The Pruzansky–Kaban classification of HFM and morphological variability of the course of bilateral IANC were accessed by two investigators independently on the coronal, cross-section, sagittal plane, and 3D images. Controls were the IANC of the unaffected sides for each patient^23^, and the variabilities of the affected sides were recorded. The recording format of the anatomical course of the IANC^17^ is shown in (Table 5), and the corresponding pictures are presented in (Fig. 1). In cases of disagreement, a craniomaxillofacial specialist with a senior professional title was invited to make the final judgment.

**Table 5:** Recording format of the anatomical course of the IANC.

Content	Recording format			
Entrance	1, Normal	2, On the top of the ramus	3, Buccal	——
Route	1, Normal	2, Bifid mandibular canal (BMC)	3, Buccal	4, Anterior aspect of the ramus
Exit	1, Normal	2, Anterior aspect of the ramus	3, Absent	4, Lingual

#### Quantitative study

**Figure 2:**
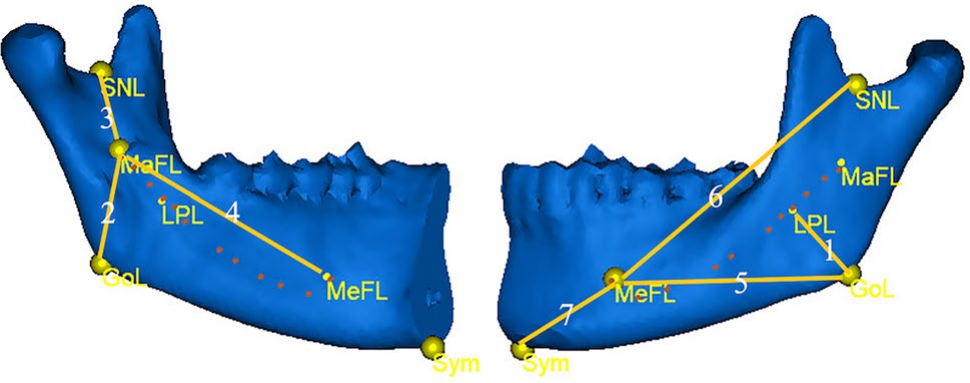
Reference points and distances in the quantitative study 1, the shortest distance between the gonion and the inferior border of the IANC (Sd). 2, mandibular foramen-gonion (MaF-Go). 3, mandibular foramen-sigmoid notch (MaF-SN). 4, mandibular foramen-mental foramen (MaF-MeF). 5, mental foramen-gonion (MeF-Go). 6, mental foramen-sigmoid notch (MeF-SN). 7, mental foramen-symphysis (MeF-Sym).

All patients had measurements of the normal and microsomic sides. Five reference points (Table 6 and Fig. 2) were selected. They were marked on 3D images and then adjusted on the coronal, sagittal, and axial images of the multiplanar reformatting setting. The software Mimics 19.0 can automatically calculate the 3D straight-line distance between two selected landmarks. Seven sets of distances^13–15^ were measured on both sides: 1. the shortest distance between the gonion and the inferior border of the IANC (Sd); 2. mandibular foramen-gonion (MaF-Go); 3. mandibular foramen-sigmoid notch (MaF-SN); 4. mandibular foramen-mental foramen (MaF-MeF); 5. mental foramen-gonion (MeF-Go); 6. mental foramen-sigmoid notch (MeF-SN); 7. mental foramen-symphysis (MeF-Sym). ﻿Each distance was measured three times over three occasions. Each unaffected side served as the control^23^.

**Table 6:** Reference points and their descriptions.

Points	Full Name	Descriptions
Sym	Symphysis	Most inferior point of the middle of the symphysis
Go	Gonion	Posterior and inferior point of the mandibular angle
SN	﻿Sigmoid notch	﻿Most concave point between the coronoid and condylar processes
MaF	﻿Mandibular foramen	﻿The middle point of the mandibular foramen fossa
MeF	Mental foramen	Most inferior point of the lower border of the mental foramen

### Ethical approval

This retrospective chart review study involving human participants was in accordance with the ethical standards of the institutional and national research committee and with the 1964 Helsinki Declaration and its later amendments or comparable ethical standards. The Human Investigation Committee (IRB) of Shanghai Ninth People’s Hospital approved this study.

### Informed consent

The need for informed consent was waived by the Ethics Committee that approved the study because of the study’s retrospective design.

### Statistical analysis

For the morphological study, the two-sided Fisher exact test was used to compare the differences between affected and unaffected sides, while the Cochran-Armitage trend test was used to analyze the relationship between the variability of IANC and the severity of mandibular deformities. For the quantitative study, the differences between the two sides were analyzed by using Student’s *t*-test: paired sample for means, and the Wilcoxon signed-rank test was used to repeat analysis in case the data didn’t follow a normal distribution. Significant levels were set as the 0.05 level and regarded *P* < 0.05 as statistically significant.

## Data Availability

Correspondence and requests for materials and data should be addressed to Y.Z.
